# Perspective: The Rapidly Expanding Need for Biosecurity by Design

**DOI:** 10.34133/2022/9809058

**Published:** 2022-05-25

**Authors:** Diane DiEuliis

**Affiliations:** National Defense University, Fort McNair, Washington, DC, USA

## Abstract

Advancing biotechnologies are revolutionizing not only health and medicine, but also many different sectors such as agriculture, energy, chemistry, and textiles. As synthetic biology is leveraged as a programmable platform for the creation and biodesign of high-value biological medicines, foods, and commodities, the world is facing new territory in terms of ensuring the safety and security of both novel and engineered biological organisms, as well as the biological and digital platforms in which they are designed. Biosecurity practices and policies have traditionally revolved around preventing the misuse of biological pathogens, primarily through controlling access to pathogens. The advent of biodesign capabilities, such as gene editors, gene synthesis capabilities, and genetic engineering, requires a reevaluation of traditional biosecurity policies to mitigate risks associated with such engineering of biological entities. Here, features of “Biosecurity by Design” approaches are described, including the application of risk/benefit analysis and risk mitigation, post-COVID opportunities, and ethical global norms in the progression of biodesign and growing bioeconomies.

## 1. Introduction

During the second annual BioDesign Conference [1], the progress of innovations in advancing biotechnologies was discussed. Such innovations, while offering benefits and advantages, also continue to emphasize the need for biosecurity awareness in their design and application. The ways and means to expand biosecurity policies to keep pace with biotechnology’s advances, while ensuring downstream benefits and avoiding the stifling of innovation and benefits, remain an important task for governments and their constituents. Here, the role of “Biosecurity by Design” is defined and described, particularly in light of the COVID-19 pandemic, as a viable approach to mitigating risk, while still realizing the benefits of biotechnology to society.

## 2. Traditional Biosecurity Increasingly Falls Short

Traditionally, biosecurity policy has been utilized across a broad biological risk spectrum, from the diversion of lawful biological research that is dual use in nature (i.e., nefarious use of the biological sciences) to protecting against accidental harms which may come from biological experimentation. These traditional biosecurity policies have focused on keeping certain biological tools and materials out of the hands of potentially bad actors; examples include the US Select Agent Program [2] and the broader Australia Group’s designated security lists of concern [3]. These polices were focused on denying access to potentially harmful biological tools and agents from the bad actors that might try to utilize them. However, such “deterrence by denial” is becoming a less viable strategy in today’s world where the ability to create and design novel biological organisms and systems is possible. Some biosecurity policies that have stemmed from these traditional biosecurity lists are successfully expanding to encompass newer technologies such as DNA synthesis, for example, where tools for screening customers and the genetic sequences they order are being successfully employed as best practices on a global scale [4]. But more creative policies and governance will be needed as biotechnologies continue to advance and create substantive global bioeconomies based on biological engineering and manufacturing. Ideally, such governance will not be one size fits all or list-based, but rather tailored to specific, identified risks, and thus “by design.” For example, before performing any dual use “gain of function,” bioengineering on pathogens, researchers should determine if there are alternative experiments which achieve the experimental goal without taking a dual use risk, such as use of nonpathogens or performing research queries in silico prior to wet bench research. Such consideration provides mitigations in cases of pathogen research but may not apply to other bioengineering scenarios.

In a study of the U.S. bioeconomy, novel risks were identified that go beyond the traditional biosecurity realm [5]. Risks associated with industrial adoption of biology to produce high value consumer products around the world include typical risks seen in other sectors. Theft, espionage, trade barriers, and international data constraints represent some of the risks that the development of biotechnology products will face, and best practices will need to be developed to address them. Further, given that bioengineering is heavily reliant on genomic and other datasets, and laboratory and biomanufacturing platforms are increasingly automated, this introduces certain cyber risks. Cybersecurity as applied to the life sciences writ large is currently a blind spot in existing biosecurity governance [6]. Described as “digital biosecurity,” this is also a component of Biosecurity by Design intended to mitigate the very specific risks associated with cyber physical systems and datasets, such as hacking, infiltration, or disruption of biological research or platforms. In the U.S., a new collaborative organization has been created as a resource for laboratories and companies alike, to address unique cyber threats to the bioeconomy, while facilitating a secure industry [7]. As the bioeconomy increasingly adopts and relies on artificial intelligence (AI) and “big data” tools, it will be important to apply risk assessments at the beginning of tool development. For example, a recent algorithm for identifying novel pharmaceuticals was created using publicly available chemical pathway datasets [8]. This same algorithm could be used to identify chemical weapon entities such as nerve agents—but this feature was only demonstrated after the data tool was created and in use [9]. Moreover, it will be important address issues of inequity, privacy, power, and personal autonomy into early risk assessment frameworks. For example, it has already been shown that some AI models in public health reflect inherent or societal bias which should be mitigated [10].

The core tenet of a Biosecurity by Design approach is the practice of risk/benefit assessments, followed by the application of directed risk mitigation strategies. The most effective biosecurity policies are those which do not stifle innovation but rather ensure awareness of risks so that mitigations can be put in place which minimize risk and maximize benefit. Prior to COVID-19, at the Meeting of Experts on Review of Science and Technology (during the 2019 meeting of the Biological Weapons Convention), discussions highlighted the available international tools and best practices for risk/benefit assessments that should increasingly become norms for biodesigners. The paper, “Approaches to Risk and Benefit Assessment for Advances in the Life Sciences” (submitted by the United States of America) emphasized building a “toolkit” that could include standards, checklists, risk assessment frameworks, mitigation approaches, continuous monitoring, norms, and guidance [11].

## 3. Biodesign Opportunities in the Post-COVID World Can Support Biosecurity

In the aftermath of the COVID-19 pandemic, it has been observed that better biosecurity policies could have assisted with more rapid responses to understand the pandemic’s origins. Analysis of the full scope of biosecurity policies reveals technical and organizational gaps in the ability to do forensics and attribution [12] on a global scale that could have assisted with more rapidly uncovering the origins of emerging infectious diseases such as COVID-19. The U.S. has funded several programs [13] which offer proof of principle for forensics—these could become essential or standardized tools that are shared internationally to address the forensics challenge. In addition, a network of international labs, called “RefBio” [14], has been created and could support the UN or other efforts in future pandemics.

Global Supply Chains were also dysfunctional during COVID-19 and offer an area of technical opportunity for biodesign. For example, DNA plasmids or mRNA vaccine prototypes based on regularly gathered genomic biosurveillance data could be created to support rapid vaccine or antibody therapy designs very early in outbreak scenarios. Also, material biodesign could offer novel approaches for the production of raw materials for nonmedical countermeasures to include resins, polyprolylene, or nitrile butadiene rubber [15]. If these materials could be designed and made on biobased platforms, such platforms could be distributed throughout many countries to provide needed materials in the manufacture of gloves, vials, masks, and other critical needs for pandemic response.

## 4. Public Perception and the Ethics Should Be Core Considerations in Biosecurity by Design

Assessing public perceptions and values is an important aspect of biosecurity and should be part of design strategies for biotechnological products for public use. As bioeconomies grow in many countries, assessing the ethical, legal, and social implications (ELSIs) will be essential. A 2016 Pew study found that while many individuals would adopt novel biotechnologies in the interest of health and medicine, they are less trusting of biotechnologies for purposes outside of disease treatment [16]. A more recent study found even greater concerns at the intersection of biotechnology and AI, to include those of privacy, autonomy, or equitable benefits to all in society [17]. These studies emphasize that ethical, legal, and social implications (ELSIs) of emerging biotechnology should be a core feature of Biosecurity by Design as applied early in technology development. Some international, collaborative ELSI programs have been initiated, such as “elsi2.0” [18]. Such programs, along with the robust discussion of ELSI issues during the BioDesign Conference itself, offer promise moving forward.

Moreover, given the environmental implications of many biodesigns, this dimension should be added to traditional ELSI studies (thus denoted as ELSEIs). For example, in the U.S., efforts are underway to the biodesign of a more a resilient chestnut tree, resistant to fungal blight that has depleted the species through the country. Renewal of the chestnut tree to its prior distribution could enable the removal of megatons of carbon dioxide from the atmosphere, but it may also have impacts on ecosystems, invasiveness, and other species protections. Similarly, there are ELSEI considerations for engineered probiotics: how should they be regulated, is the release of microbes via gut excretion into the environment safe? How can protection against potential weaponization be provided? These provide just a few examples of the varied needs for biosecurity that should be tailored for these individual scenarios.

## 5. Conclusions

The annual BioDesign Conference has offered a unique opportunity to discuss issues of Biosecurity by Design to a global audience of biodesigners interested in ensuring the responsible use of biotechnology. Suggestions for designing better biosecurity policy could include the international development of risk/benefit assessment frameworks; a focus on biodesign solutions for public health and other global needs during pandemic response, and engagement with communities on biotechnology applications with ELSEI. Use of these tools can assure the benefits of biotechnology are safe, secure, and fully realized.
